# Atypical Presentation of Acute Compartment Syndrome in the Lower Limb: A Case Report of When Pain Does Not Guide the Diagnosis

**DOI:** 10.7759/cureus.87488

**Published:** 2025-07-07

**Authors:** José C González-Rodríguez, Maria Cristofori, Emmanuel E Cortés-Marín, José A Antunez Oliva, Pablo Alvarez Aguilar

**Affiliations:** 1 Internal Medicine, Universidad de Costa Rica, San José, CRI; 2 General Medicine, Universidad de Ciencias Médicas (UCIMED), San José, CRI; 3 General Medicine, Universidad de Costa Rica, San José, CRI; 4 Critical Care Medicine, Universidad de Costa Rica, San José, CRI

**Keywords:** atypical presentation, clinical diagnosis, compartment syndrome, fasciotomy, polytrauma

## Abstract

Acute compartment syndrome (ACS) is a surgical emergency that, if not promptly treated, can lead to muscle necrosis, limb loss, or death. Although disproportionate pain is traditionally considered the hallmark symptom, atypical presentations without pain have been described. We report the case of a 42-year-old male who sustained multiple traumatic injuries in a motorcycle-vehicle collision and subsequently developed silent compartment syndrome in the left thigh and leg. Despite being conscious and neurologically intact, the patient did not report significant pain at any point. The diagnosis was suspected based on objective findings, including tense edema, absent distal pulses, and progressive motor dysfunction. Invasive compartment pressure measurements confirmed the diagnosis, leading to urgent fasciotomies and intensive care management, including renal replacement therapy. This case highlights the importance of maintaining a high index of suspicion for ACS in patients with major trauma, even in the absence of pain, and supports early intervention to prevent irreversible sequelae.

## Introduction

Acute compartment syndrome (ACS) is a medical emergency characterized by increased pressure within a closed osteofascial compartment, compromising tissue perfusion and potentially leading to ischemia, muscle necrosis, nerve dysfunction, and even limb loss or death if not promptly recognized and treated [1]. In clinical practice, the earliest and most reliable diagnostic sign is pain that is disproportionate to the inciting stimulus and unrelieved by conventional analgesia [1,2]. However, this hallmark feature is very rarely absent or underestimated in specific contexts, such as altered mental status, posing a significant diagnostic challenge [3].

While most ACS cases occur in conscious patients who report severe pain, the literature describes situations in which pain may be absent or inadequately expressed, such as in sedated patients, those with altered mental status or concomitant neurological injury, or, as in the present case, even in conscious individuals with an insidious and non-specific clinical course. This condition, which could be termed *silent* compartment syndrome, represents an underrecognized diagnostic threat and may delay definitive surgical intervention [3,4]. When left untreated, ACS can progress to massive necrosis, sepsis, multiorgan failure, and even death, with reported mortality rates of up to 47% in critically ill patients, especially when associated with polytrauma and rhabdomyolysis [5].

In the setting of polytrauma, where multiple injuries compete for immediate clinical attention, and where post-traumatic edema and external immobilization devices may obscure compartmental evolution, diagnosis becomes even more challenging. The absence of disproportionate pain does not exclude the diagnosis, and clinicians must maintain a high index of suspicion based on objective signs such as tense compartments, pallor, coolness, motor impairment, and absence of distal pulses. In such cases, invasive measurement of intra-compartmental pressure may serve as a crucial tool to confirm the diagnosis and guide timely surgical decisions [4,6].

This report describes the case of a young male polytrauma patient who developed ACS of the left thigh and leg with no prominent pain or classical clinical signs. His course required extensive fasciotomies, renal replacement therapy, and intensive surgical management. Through this case, we aim to highlight the importance of recognizing atypical presentations of ACS and to emphasize the value of continuous clinical surveillance in high-risk patients, even in the absence of pain.

## Case presentation

The patient was a 42-year-old man with no known medical history who had been transferred to a tertiary care hospital from a lower-level facility after being involved in a traffic accident involving a collision between a motorcycle and a light vehicle. Upon arrival at the Emergency Department, the patient was conscious, alert, and oriented with a Glasgow Coma Scale score of 15. Initial vital signs showed tachycardia and a mean arterial pressure of 70 mmHg. He exhibited a normal respiratory pattern, with an oxygen saturation of 97% on room air. His chief complaint was pain in both lower extremities and the left upper extremity.

**Figure 1:**
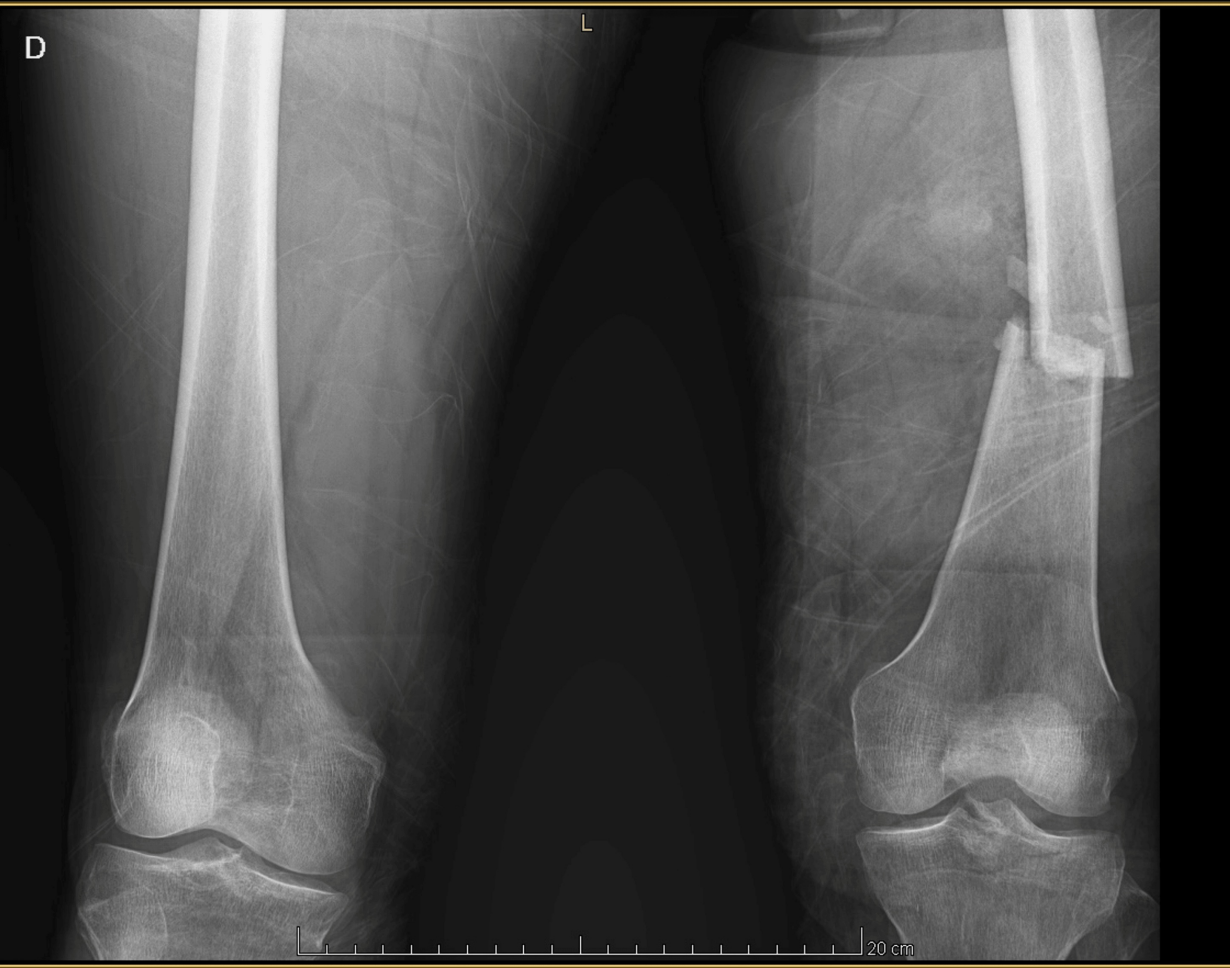
Bilateral femur X-ray showing a left femoral fracture. Anteroposterior radiograph of both femurs showing a transverse fracture of the midshaft of the left femur, without associated displacement. This injury was part of a multiple trauma scenario and contributed to the development of acute compartment syndrome.

Upon initial trauma assessment, the patient presented with polytrauma involving multiple extremities. The left upper extremity sustained a traumatic partial hand amputation associated with an open carpal fracture, classified as Gustilo-Anderson grade IIIB. The left lower extremity presented with multiple open fractures, including a Gustilo-Anderson grade II open femoral fracture (Figure 1), a traumatic knee arthrotomy (a breach of the joint capsule due to injury), and a Gustilo-Anderson grade II open tarsal fracture. The right lower extremity exhibited a severe open injury to the forefoot with extensive soft tissue loss, resulting in exposure of the metatarsal heads and proximal phalanges. Despite the severity of the orthopedic injuries, vascular assessment via Doppler examination of all affected limbs revealed no evidence of arterial injury at the time of evaluation.

The patient was taken to the operating room for surgical wound irrigation and debridement. During the procedure, additional interventions included a left transradial amputation, closed reduction with external fixation of the femoral fracture, and primary closure of other soft tissue compromising injuries. No clinical signs of compartment syndrome were observed intraoperatively. He was subsequently transferred to the Surgical Intensive Care Unit (SICU) for postoperative monitoring and multidisciplinary management.

Upon admission to the SICU, the patient did not report pain, and an analgesic regimen was initiated consisting of intravenous paracetamol and continuous intravenous lidocaine infusion. Early in the clinical course, the patient developed KDIGO (Kidney Disease: Improving Global Outcomes) stage 3 acute kidney injury, accompanied by laboratory findings indicative of rhabdomyolysis.

Systemically, the patient required intensive management, including a massive transfusion protocol, initiation of renal replacement therapy with intermittent hemodialysis, and aggressive fluid resuscitation. Empirical broad-spectrum antibiotic therapy was initiated with cefotaxime and clindamycin, later escalated to linezolid, in view of the high risk of infection in ischemic tissues.

Despite the absence of severe pain or other classical symptoms of compartment syndrome, the patient developed progressive swelling of the left thigh and leg, along with stiffness, coldness, and absence of distal pulses. Sensory loss in the left lower extremity was noted, with a gradual and ascending pattern.

**Figure 2:**
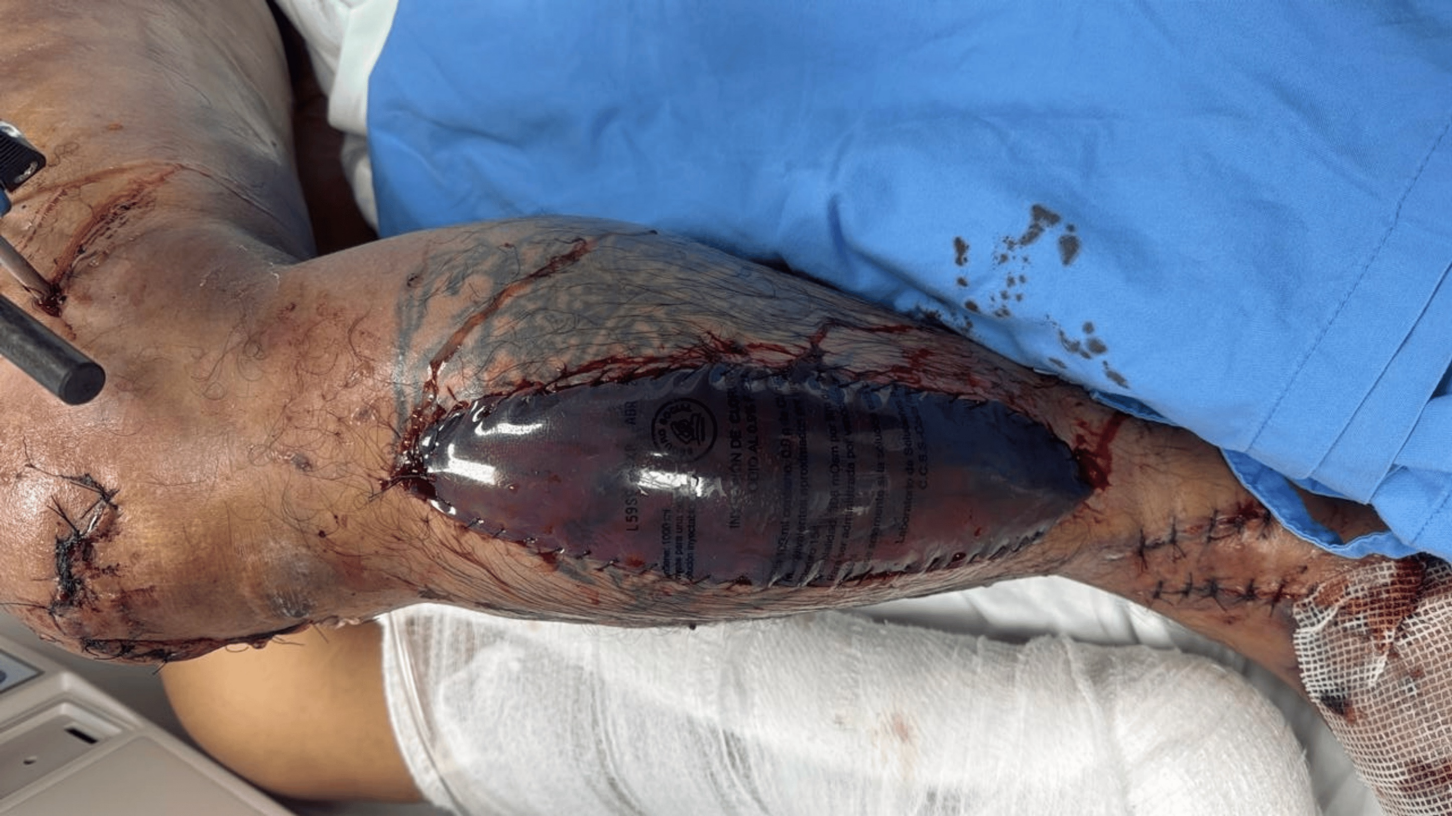
Anterolateral fasciotomy of the left leg.

These findings prompted an urgent vascular reassessment. Deep vein thrombosis was ruled out as a differential diagnosis, and the patient was closely monitored. Invasive compartment pressure measurements were subsequently performed in the distal left leg, revealing values of 18 mmHg in the anterior compartment, 30 mmHg in the superficial posterior compartment, and 28 mmHg in the deep posterior compartment. These values were obtained in the context of a diastolic blood pressure ranging from 60 to 70 mmHg. Given the ongoing clinical deterioration and signs of impaired distal perfusion, a diagnosis of compartment syndrome was made, and the patient was taken emergently to the operating room. A left leg anterolateral fasciotomy (Figure 2) and medial fasciotomy were performed and covered using a Bogota bag.

**Figure 3:**
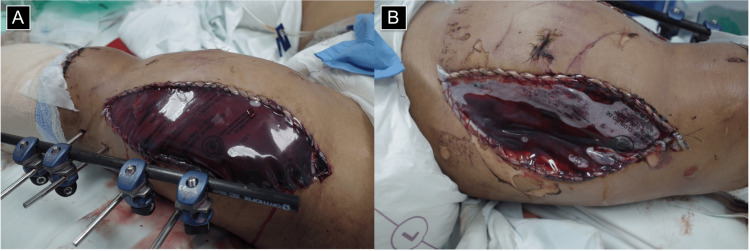
Decompressive fasciotomies of the left thigh in acute compartment syndrome. (A) Anterolateral fasciotomy exposing the anterior compartment, with external fixation for associated femoral fracture. (B) Posteromedial fasciotomy for release of the posterior and medial compartments. Both wounds were left open and covered with sterile transparent dressings for second-look surgery.

Due to the need for further surgical debridement and persistent clinical findings suggestive of compartment syndrome, now extending to the thigh, the patient underwent reoperation three days later. During this procedure, a left thigh fasciotomy and additional wound irrigation were performed. The outcome of the intervention is shown in Figure 3. Postoperatively, effective decompression was achieved, with preservation of viable tissue.

The patient remained under management in the SICU, where he continued to receive renal support therapy and underwent surgical wound irrigation every 48 to 72 hours, until clinical resolution was achieved and definitive wound closure could be considered.

## Discussion

ACS is a surgical emergency that occurs when the increase in interstitial pressure within a closed osteofascial compartment exceeds capillary perfusion pressure, leading to tissue hypoxia, progressive ischemia, muscle necrosis, and, if left untreated, irreversible functional loss, limb amputation, or even death [2]. This condition is most frequently observed in the context of orthopedic trauma, particularly in the lower extremities, and is commonly associated with diaphyseal fractures, crush injuries, burns, or reperfusion syndromes following prolonged ischemia [7,8].

The clinical syndrome was first described in 1881 by German surgeon Richard von Volkmann, who observed ischemic contractures in children following inadequately managed fractures [9,10].

The diagnosis of ACS remains primarily clinical, although it presents significant challenges in polytraumatized, sedated, or neurologically impaired patients. The most classic and sensitive symptom historically described is pain that is disproportionate to the stimulus and exacerbated by passive stretching [1,11]. However, this sign may go unnoticed in certain clinical contexts due to factors such as analgesia, altered level of consciousness, or associated neuropathies. This variability in clinical presentation has led to the identification of a specific subtype known as *silent compartment syndrome*, in which pain is not a predominant feature, and diagnosis relies on other physical or functional findings [4].

Although silent presentations of compartment syndrome are rare, they have been documented in the literature for several decades. In 1989, Wright et al. described a case of *occult compartment syndrome*, in which the patient, despite being awake and oriented, exhibited no significant pain but developed compartmental ischemia with severe intraoperative findings [12]. This type of presentation highlights that the absence of pain does not exclude the diagnosis, and that clinical deterioration may progress insidiously to a critical state if not promptly addressed [1,8,13].

The case presented herein falls within this unusual category. Our patient, despite being conscious and neurologically intact, did not report significant pain at any time, neither during the initial emergency department stay nor during the early hours in the SICU. Clinical suspicion was based on objective signs such as tense edema, tissue firmness, absence of distal pulses, and progressive loss of functional mobility, all within a high-risk post-traumatic context, prompting urgent surgical exploration. This evolution is consistent with the few published cases with similar characteristics, in which pain was either absent or of limited diagnostic value [3,4,13-15].

In scenarios involving ambiguous or limited clinical evaluation, such as unconscious, intubated, or deeply sedated patients, objective tools like compartment pressure measurement have been proposed [16].

The concept of elevated compartment pressure as a central component in the pathophysiology of ACS was first introduced by Matsen in 1975 [10,17]. Subsequently, a technique for invasive measurement of intra-compartmental pressure was developed, establishing surgical intervention criteria based on a delta pressure (diastolic pressure minus intra-compartmental pressure) of less than 30 mmHg [10,18]. Currently, compartment pressures greater than 30 mmHg are also considered pathological [6]. Nevertheless, compartment pressure measurement is only one element in the diagnosis of ACS and should not be used indiscriminately or as the sole criterion. Some studies have reported false positive rates of up to 35%, particularly in patients without clear clinical manifestations or when using poorly calibrated devices or outside the appropriate physiopathological context [19].

Other complementary diagnostic modalities have been proposed, such as ultrasonography and near-infrared spectroscopy, among others. Similarly, the absence of the classic clinical picture does not rule out a diagnosis of ACS in patients with predisposing factors [16,18,20].

Delayed diagnosis or treatment of ACS carries catastrophic consequences. Progression to extensive muscle necrosis favors the development of deep infections, sepsis, rhabdomyolysis, and multiorgan failure. Retrospective studies have estimated a mortality rate of up to 47% when compartment syndrome is not recognized or treated in a timely manner, especially in patients with severe polytrauma and associated organ dysfunction [5]. Even among survivors, morbidity remains significant, including amputations, contractures, neuropathies, and prolonged functional disability [1,2,5].

This case underscores the importance of maintaining a high index of suspicion for compartment syndrome in patients with severe orthopedic trauma, even in the absence of typical pain. Careful monitoring for indirect signs, such as tense edema, progressive motor impairment, diminished peripheral pulses, or loss of active mobility, should alert the treating team, particularly in high-risk settings [4]. The implementation of serial evaluation protocols, along with continuous training of clinical and surgical personnel, is essential for the timely recognition of atypical presentations. Early intervention with wide fasciotomies, even in the presence of diagnostic uncertainty, may be the difference between preserving a functional limb and facing amputation or permanent disability [3,6]. In this context, a coordinated multidisciplinary approach, integrating surgical, intensive care, and rehabilitation teams, is critical to improving both functional and survival outcomes in these patients [4,6-8,10,13].

## Conclusions

ACS remains a medical and surgical emergency in which time is a critical factor determining the difference between functional recovery and irreversible limb loss. This case illustrates an atypical presentation in a conscious patient without predominant pain, in whom clinical suspicion was based on the progression of physical signs rather than classical findings. The experience highlights the need to recognize that ACS may present silently, and that pain, although historically considered the most sensitive symptom, is not universal. This unreliability of subjective symptoms underscores the critical importance of using alternative, objective diagnostic methods, such as intra-compartmental pressure measurement, to confirm suspicion when clinical signs are equivocal or atypical. Employing these tools provides quantifiable data that can reduce delays to definitive surgical intervention. In this context, clinical judgment, strict monitoring, the use of objective measurements when indicated, and the willingness to perform fasciotomy without waiting for the full constellation of traditional symptoms are essential.
